# *HtpG* contributes to *Salmonella* Typhimurium intestinal persistence in pigs

**DOI:** 10.1186/s13567-015-0261-5

**Published:** 2015-10-14

**Authors:** Elin Verbrugghe, Alexander Van Parys, Bregje Leyman, Filip Boyen, Freddy Haesebrouck, Frank Pasmans

**Affiliations:** Department of Pathology, Bacteriology and Avian Diseases, Faculty of Veterinary Medicine, Ghent University, Merelbeke, Belgium; Department of Clinical Chemistry, Microbiology and Immunology, Ghent University, Ghent, Belgium; Department of Nutrition, Genetics and Ethology, Faculty of Veterinary Medicine, Ghent University, Merelbeke, Belgium

## Abstract

*Salmonellaenterica* subspecies *enterica* serovar Typhimurium (*Salmonella* Typhimurium) contamination of pork, is one of the major sources of human salmonellosis. The bacterium is able to persist and hide in asymptomatic carrier animals, generating a reservoir for *Salmonella* transmission to other animals and humans. Mechanisms involved in *Salmonella* persistence in pigs remain poorly understood. In the present study, we demonstrate that the *SalmonellahtpG* gene, encoding a homologue of the eukaryotic heat shock protein 90, contributes to *Salmonella* Typhimurium persistence in intestine-associated tissues of pigs, but not in the tonsils. *HtpG* does not seem to play an important role during the acute phase of infection. The contribution to persistence was shown to be associated with *htpG*-dependent *Salmonella* invasion and survival in porcine enterocytes and macrophages. These results reveal the role of HtpG as a virulence factor contributing to *Salmonella* persistence in pigs.

## Introduction

Salmonellosis is regarded as one of the most important bacterial zoonotic diseases [1, 2]. The main route of human infection is through the consumption of contaminated food, such as pork, with *Salmonellaenterica* subspecies *enterica* serovar Typhimurium (*Salmonella* Typhimurium) being the most frequently isolated serovar from slaughter pigs [3]. *Salmonella* Typhimurium seldom produces systemic infections in healthy adult animals. The bacterium is however able to colonize the alimentary tract and may cause acute enteritis followed by persistence. The ability to cause a persistent colonization in the host is a major characteristic of *Salmonella* virulence [4]. Persistence is here defined as a chronic presence of *Salmonella* Typhimurium in the porcine host. In our infection model [5] as well as in practice, this is characterized by relatively high level infection loads in tonsils but low intestinal loads resulting in intermittent faecal shedding. Persistently colonized carrier animals are difficult to distinguish from uninfected pigs and they constitute a continuous reservoir of *Salmonella* bacteria. During periods of stress, like transport to the slaughter house, a flare up of this asymptomatic colonization may occur [6]. Until now, the mechanism of prolonged colonization in carrier pigs remains poorly known, which seriously hampers the development of efficient mitigation measures. A thorough understanding of how *Salmonella* is able to persist requires the identification of bacterial genes involved.

Recently, using in vivo expression technology (IVET), Van Parys et al. identified 37 *Salmonella* Typhimurium genes that are specifically expressed during persistence in pigs [7]. Although these are potential virulence genes, their individual contribution to *Salmonella* persistence remains to be further explored. In the present study, we focused on one of these 37 genes, the *htpG* gene that encodes a homologue of the eukaryotic heat shock protein 90 (hsp90), a chaperone being important for the maintenance, activation, stabilization or maturation of proteins [8]. The aim of this study was to define the role of this gene during colonization and persistence of *Salmonella* Typhimurium in pigs.

## Materials and methods

### Bacterial strains

*Salmonella* Typhimurium strain 112910a phage type 120/ad, isolated from a pig stool sample, was used as the wild type strain (WT). A spontaneous nalidixic acid resistant derivative of the wild type strain was used in the in vivo experiment (*Salmonella* WT^nal^). *Salmonella* Typhimurium deletion (*Salmonella* Δ*htpG*) and kanamycin resistant substitution (*Salmonella* Δ*htpG:kanR*) mutants (Table 1) were constructed according to the one-step inactivation method as described previously [9] and slightly modified for use in *Salmonella* Typhimurium [10].

**Table 1:** **Primers used in this study to create the** ***Salmonella*** **Δ** ***htpG*** **mutants**

Application	Primers	Sequences (5′ to 3′)
Mutagenesis	*htpG* forward	CCCTCAACGTATTTTTACCATTAAAAATGGCATTGTTGAGGTCTATCCACTGTGTAGGCTGGAGCTGCTTC
Mutagenesis	*htpG* reverse	CGGATAAGACGCTTCGCGTCGCCATCCGGCAGTCAGATGAGCGTTACATATGAATATCCTCCTTAG

### Cell cultures and experiments

Invasion and intracellular survival of *Salmonella* was quantified in porcine intestinal epithelial cells (IPEC-J2) and in porcine macrophages. IPEC-J2 cells [11, 12] and primary porcine alveolar macrophages (PAM) [13] were cultured as previously described. To examine whether the ability of *Salmonella* Typhimurium to invade and proliferate in PAM and IPEC-J2 cells was altered after deletion of *htpG*, gentamicin protection assays were performed using *Salmonella* WT and *Salmonella* Δ*htpG*. Therefore, PAM and IPEC-J2 cells were seeded in 24-well plates at a density of approximately 10^6^ and 5 × 10^5^ cells per well, respectively. PAM were allowed to attach for 2 h and IPEC-J2 cells were allowed to grow for 24 h. Subsequently, *Salmonella* was inoculated into the wells at a multiplicity of infection (MOI) of 10:1. To synchronize the infection, the inoculated multiwell plates were centrifuged at 1500 rpm for 10 min and incubated for 30 min at 37 °C under 5% CO_2_. After washing the cells three times with Hank’s balanced salt solution (HBSS), they were supplemented for 1 h with fresh medium containing 100 µg/mL gentamicin to kill extracellular bacteria. After washing 3 times, the cells were lysed for 10 min with 1% Triton X-100 or 0.2% sodium deoxycholate, respectively. The number of invaded *Salmonella* bacteria was determined by plating 10-fold dilutions on Brilliant Green Agar (BGA) plates. To assess intracellular proliferation, the medium containing 100 µg/mL gentamycin was replaced after 1 h with fresh medium containing 20 µg/mL. The plates were incubated for 6 or 24 h and the number of intracellular bacteria was determined as described for the invasion.

### Role of *htpG* during persistence of *Salmonella* in pigs

The animal experiment was carried out in strict accordance with the recommendation in the European Convention for the Protection of Vertebrate Animals used for Experimental and other Scientific Purposes. The experimental protocols and care of the animals were approved by the Ethics Committee of the Faculty of Veterinary Medicine, Ghent University (EC2010/005). Thirteen four-week-old piglets (commercially closed line based on Landrace) from a serologically *Salmonella* negative breeding herd were used. The *Salmonella*-free status of the piglets was verified serologically (IDEXX, Hoofddorp, The Netherlands) and bacteriologically via repeated faecal sampling. The animals arrived at the facility 5 days before inoculation and they were housed in isolation units at 25 °C under natural day-night rhythm in HEPA-filtered stables, with ad libitum feed and water. The piglets were randomly divided in a negative control group of 3 animals and 2 groups of 5 animals that were orally inoculated with a mixture of approximately 2 × 10^7^ colony-forming units (CFU) *Salmonella* WT^nal^ and 2 × 10^7^ CFU *Salmonella* Δ*htpG:kanR*. The negative control pigs were administered 2 mL HBSS. It was our hypothesis that *htpG* contributes to persistence but that deleting this gene does not result in a lack of successful initial colonization of the pig. Based on the results of Boyen et al., we determined the *Salmonella* numbers at day 4 (acute phase) and day 21 (persistent phase) in this experiment [5]. Therefore, one group of *Salmonella*-inoculated animals was euthanized 4 days post inoculation (pi). The negative control and the other *Salmonella*-inoculated group were euthanized 3 weeks pi. Samples of the palatine tonsils, ileum and contents, ileocecal lymph nodes, cecum and contents and faeces were collected and bacteriologically analyzed as previously explained [6]. Briefly, 10% suspensions of all samples were prepared in buffered peptone water and the number of *Salmonella* bacteria was determined by plating 10-fold dilutions on BGA plates supplemented with 20 µg/mL nalidixic acid or 100 µg/mL kanamycin. Samples that were negative after direct plating but positive after enrichment in tetrathionate broth were presumed to contain 83 CFU per gram sample (detection limit for direct plating).

### Statistical analysis

All statistical analyses were performed using SPSS version 22 (SPSS Inc., Chicago, IL, USA). Normality of the data was assessed using a Kolmogorov–Smirnov and Shapiro–Wilk test. Normally distributed data were analyzed using an unpaired Student’s *t* test to address the significance of difference between mean values with significance set at *P* ≤ 0.05. If equal variances were not assessed or if the data were not normally distributed, they were analyzed using the non-parametric Kruskal–Wallis analysis, followed by a Mann–Whitney U test.

## Results

### *HtpG* plays a role during intestinal persistence of *Salmonella* Typhimurium in pigs

**Figure 1:**
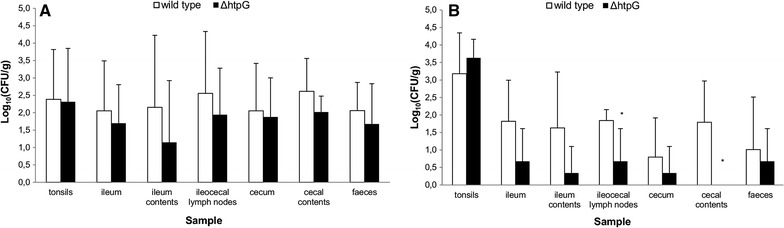
***Salmonella*** **Typhimurium** **WT** ^**nal**^ **and Δ** ***htpG:kanR*** **colonization of pigs**. Recovery of bacteria from various organs of 5 piglets orally inoculated with an equal mixture of *Salmonella* WT^nal^ and Δ*htpG:kanR*, **A** 4 days pi and **B** 21 days pi. The log 10 value of the number of CFU per gram sample is given as the mean + standard deviation. A non-parametric Mann–Whitney U test was performed and superscript (*) refers to a significant difference compared to the wild type *Salmonella* group (*p* < 0.05).

Four days pi, no significant differences were observed in the number of *Salmonella* WT^nal^ and *Salmonella* Δ*htpG:kanR* bacteria colonizing the tonsils, ileocecal lymph nodes, ileum and cecum and their contents and faeces (Figure 1A). In contrast, 21 days pi, significant differences in colonization were noticed between the two strains (Figure 1B). The *Salmonella* Δ*htpG:kanR* strain was attenuated in the intestine-associated tissues and contents: ileocecal lymph nodes (decrease of 1.2 log_10_ (CFU/g)), ileum (decrease of 1.1 log_10_ (CFU/g)), cecum (decrease of 0.5 log_10_ (CFU/g)), ileum contents (decrease of 1.3 log_10_ (CFU/g)), cecal contents (decrease of 1.8 log_10_ (CFU/g)) and faeces (decrease of 0.3 log_10_ (CFU/g)). These differences were significant in the ileocecal lymph nodes (*p* = 0.042) and cecal contents (*p* = 0.018).

### *HtpG* is involved in invasion and intracellular replication of *Salmonella* in macrophages

**Figure 2:**
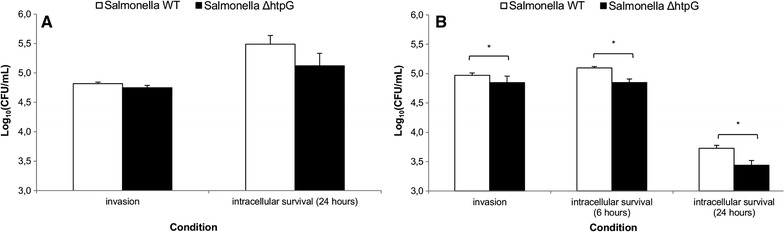
**Role of** ***htpG*** **during invasion and intracellular replication of** ***Salmonella*** **in host cells.** Number of intracellular *Salmonella* WT or *ΔhtpG* bacteria after invasion or intracellular replication (6 and/or 24 h) in **A** IPEC-J2 cells or **B** primary macrophages. The log_10_ values of the number of gentamicin protected bacteria + standard deviation are shown. Results are presented as a representative experiment conducted in sixfold. An independent t-test was performed and superscript (*) refers to a significant difference (*p* < 0.05).

In IPEC-J2 cells, we observed a tendency (*p* < 0.1) towards reduced invasion (decrease of 0.1 log_10_ (CFU/mL)) and intracellular survival for 24 h (decrease of 0.4 log_10_ (CFU/mL)) of *Salmonella* Δ*htpG* compared to the WT strain (Figure 2A). Moreover, the *htpG* gene was also shown to be important (*p* < 0.05) for *Salmonella* invasion (decrease of 0.1 log_10_ (CFU/mL)) and survival capacities at 6 h pi (decrease of 0.2 log_10_ (CFU/mL)) and 24 h pi (decrease of 0.3 log_10_ (CFU/mL)) in primary macrophages, in vitro (Figure 2B).

## Discussion

The pathogenicity of *Salmonella* has been extensively studied, both in vitro and in vivo, using different animal models like the mouse, pig and chicken. Especially the mouse model is a widely used paradigm for studying the pathogenesis of systemic disease caused by *Salmonella*. However, investigations concerning food safety should employ natural hosts to examine gastrointestinal colonization by *Salmonella*. A major source of *Salmonella* contamination of pork meat is cross-contamination in the slaughterhouse. A thorough understanding of the molecular mechanisms that *Salmonella* Typhimurium exploits to persist in the tonsils, gut and gut-associated lymphoid tissues, might contribute to the development of appropriate measures to minimize *Salmonella* contamination of porcine carcasses. Therefore, in the past decade, research has focused on the identification of virulence mechanisms contributing to its persistence in pigs. However, up to date, few data are available concerning *Salmonella* persistence in pigs [7, 14–17].

Persistence of *Salmonella* is an intriguing characteristic of the bacterium that allows maintenance of the pathogen in a host population. After bacterial invasion in hosts, such as pigs, the immune system will respond to clear the infection and bacterial survival strategies for persistence will become important. We now showed that the *htpG* gene encodes such a survival mechanism. Deletion of the *htpG* gene does not affect the acute phase of infection, but reduces the survival capacity of the bacterium in intestine-associated tissues of pigs. The specific role of HtpG in bacterial pathogenesis is largely unknown, but it is a homolog of the eukaryotic chaperone hsp90, being important in the adaptation of *Salmonella* to stress conditions [18]. During persistence in a certain host, *Salmonella* has to deal with numerous stresses, like the host’s immune system, decreased oxygen tension, nutrient limitation and starvation and shift in temperature and pH. Possibly, the HtpG chaperone helps *Salmonella* dealing with stressful events during persistence, in vitro and in vivo, leading to an increased survival of the bacterium.

In the past, using IVET, Van Parys et al. screened the tonsils, ileum and ileocecal lymph nodes for genes being induced during the persistent phase of colonization [7]. They showed that the *htpG* gene is expressed in all three organs [7]. However, we now demonstrated that the *htpG* gene plays a role during persistence of the bacterium in intestine-associated tissues of pigs but not in the tonsils. In contrast to the gut and gut-associated lymphoid tissue, *Salmonella* resides largely in an extracellular niche in the tonsils [19]. Therefore, virulence mechanisms necessary for cell invasion and intracellular survival do not contribute to tonsillar colonization and persistence [17, 20]. We also showed that *Salmonella* Typhimurium lacking *htpG* was impaired in invasion and intracellular replication in epithelial cells and macrophages. Invasion of gut epithelial cells and the capacity to survive and replicate intracellularly in macrophages are major factors contributing to intestinal *Salmonella* Typhimurium persistence in pigs. Our findings, therefore, confirm the earlier hypothesis that different molecular mechanisms are involved in *Salmonella* colonization of and persistence in the tonsils on one hand, and in the gut and gut-associated lymphoid tissue on the other hand [17, 19, 20]. Although *Salmonella* persistence in tonsils represents a major factor in persistence of the bacterium in pigs, the bacterial genes involved remain largely unknown.

Based on the results obtained by Boyen et al., we sacrificed pigs 3 weeks post inoculation and used this model to examine *Salmonella* persistence in pigs [5]. Although its contribution to long-term (several months) persistence is not clear, we can conclude that *htpG* contributes to *Salmonella* Typhimurium persistence in the porcine intestine until 3 weeks after inoculation.
